# Ivabradine use in pregnant women—treatment indications and pregnancy outcome: an evaluation of the German Embryotox database

**DOI:** 10.1007/s00228-020-03066-w

**Published:** 2021-01-26

**Authors:** Maria Hoeltzenbein, Marie-Louise Lehmann, Evelin Beck, Katarina Dathe, Christof Schaefer

**Affiliations:** Pharmakovigilanz- und Beratungszentrum für Embryonaltoxikologie, Institute of Clinical Pharmacology and Toxicology, Charité – Universitätsmedizin Berlin, corporate member of Freie Universität Berlin, Humboldt-Universität zu Berlin, and Berlin Institute of Health, Augustenburger Platz 1, D - 13353 Berlin, Germany

**Keywords:** Ivabradine, Pregnancy, Reproductive age, Drug safety, Tachycardia

## Abstract

**Purpose:**

Ivabradine has been approved for the treatment of chronic heart failure and chronic stable angina pectoris in Europe. Based on adverse outcomes of reproductive animal studies and the lack of human data, ivabradine is considered contraindicated during pregnancy. The aim of this observational study is to analyse ivabradine use before and during pregnancy.

**Methods:**

We evaluated all ivabradine-related requests to the German Embryotox Institute from 2007 to 2019. Exposed pregnancies were analysed as to their outcome.

**Results:**

Off-label use for supraventricular tachycardia was frequent in women of childbearing age. Of 38 prospectively ascertained pregnancies with ivabradine exposure and completed follow-up, 32 resulted in live births, 3 in spontaneous abortions, and 3 were electively terminated. One neonate presented with major birth defects (atrial septal defect and cleft palate). In 33/38 patients, ivabradine was discontinued after confirmation of pregnancy without cardiac deterioration and 5/38 women continued ivabradine throughout pregnancy. In addition, there were 3 retrospectively reported pregnancies including one major birth defect (tracheal atresia).

**Conclusion:**

This case series represents the largest cohort of ivabradine-exposed pregnancies, published so far. According to our findings, ivabradine appears not to be a major teratogen. However, established drugs of choice with strong evidence of low risk for the unborn should be preferred in women planning pregnancy. After inadvertent exposure during pregnancy or lack of treatment alternatives, fetal ultrasound for structural anomalies and growth restriction is recommended. In addition, close monitoring is necessary in pregnant women with supraventricular arrhythmias or cardiac disease.

## Introduction

Ivabradine is a heart rate-lowering agent, acting through selective inhibition of the If (I-funny) channel without additional hemodynamic effects on cardiac function. The approved treatment indications in Europe are chronic heart failure and symptomatic treatment of chronic stable angina pectoris after failing or intolerance of beta-blockers [1].

Preclinical studies have suggested fetotoxicity and teratogenic effects. In pregnant rats, ivabradine exposure equivalent to therapeutic levels in humans was associated with increased intrauterine and postnatal mortality. Ventricular septal defects and complex anomalies of the great arteries were noted at doses 3 times the therapeutic human exposure. In pregnant rabbits, reduced fetal and placental weight as well as ectrodactyly was observed at doses 15 times the therapeutic human exposure [2]. In addition, dose-dependent mortality was seen in chicken and mice embryos [3, 4].

ESC guidelines consider ivabradine contraindicated during pregnancy and recommend discontinuation independent of treatment indication before conception with close clinical and echocardiographic monitoring [5].

Published data on ivabradine-exposed pregnancies are limited to three case reports, one with ivabradine treatment in the late first and early second trimester for sinus tachycardia after myocardial infarction [6], and the second starting ivabradine in the second trimester for deteriorating tachycardia-induced cardiomyopathy [7]. In the third case with first trimester exposure, pregnancy was electively terminated [8].

The limited human experience along with the teratogenic effects in animal studies makes it difficult to counsel inadvertently exposed pregnant women and their health care providers (HCP). Therefore, we decided to evaluate ivabradine-exposed pregnancies recorded by the German Embryotox Institute.

## Methods

### Study design

The German Embryotox Institute offers risk assessment on drug exposure during pregnancy to HCPs and patients. Up to 15,000 annual requests are answered, and in approximately 3500 critically exposed cases per year, the course and outcome of pregnancy are documented. At the initial contact, maternal characteristics and detailed exposure assessment are asked for after informed consent. About 8 weeks after the estimated date of birth, information on course and outcome of pregnancy is collected via structured telephone interview and mailed questionnaires. After a case by case plausibility check, additional information and health care records are requested in cases of adverse outcomes or inconsistent information.

Weeks of pregnancy were based on ultrasound and if not available on the last menstrual period (LMP). First trimester was defined as gestational week 2 + 0 (conception) until 12 + 6. Major birth defects were classified according to EUROCAT [9]. In prospectively ascertained pregnancies, neither the outcome of pregnancy nor prenatal pathology was known at the time of first contact. Pregnancies that were reported after birth or because of prenatal pathology were considered retrospective and evaluated separately. A detailed description of methodology adapted to the recommendations of the Strengthening of the Reporting of Observational studies in Epidemiology (STROBE) statement is given in Schaefer et al. [10] and Dathe and Schaefer [11].

We evaluated all requests on ivabradine to the German Embryotox Institute from 2007 to 2019.

### Statistical analyses

Descriptive statistics were applied. For age, BMI, gestational weeks, neonatal weight, length, and head circumference, median and interquartile ranges are presented. Birth weights were adjusted to sex and gestational age at birth and percentile categories and standard deviation scores (SDS) were calculated according to the German perinatal survey [12].

## Results

**Fig. 1:**
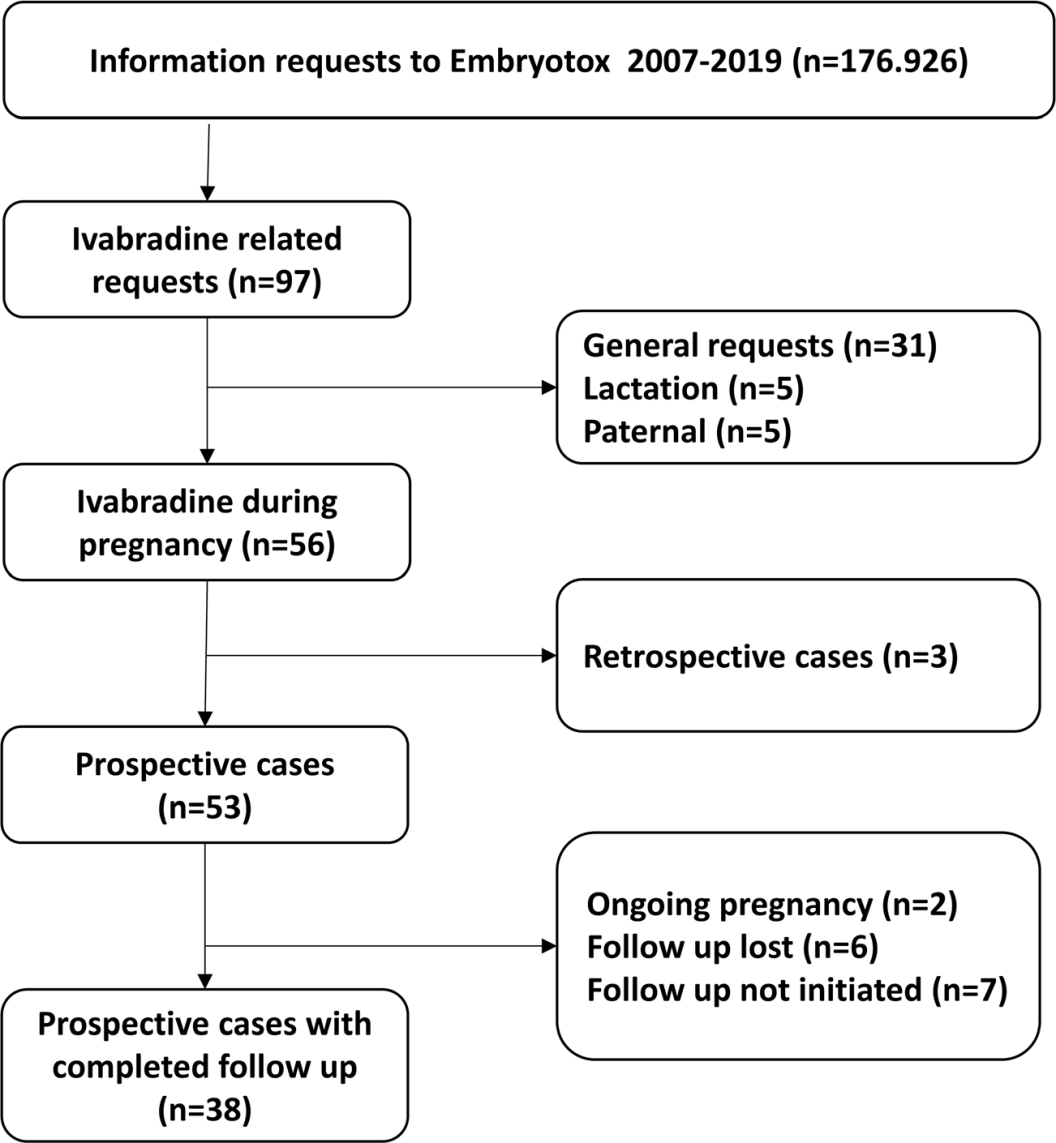
Overview on information requests to the German Embryotox institute on ivabradine

**Fig. 2:**
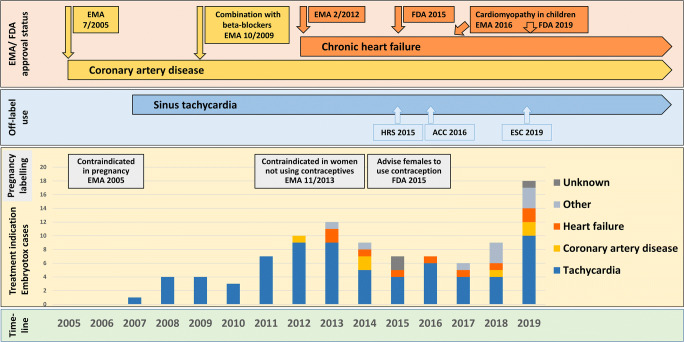
Treatment indication for ivabradine in 97 Embryotox requests over time in relation to pregnancy labelling and approval status by EMA/FDA. Recommendations for use of ivabradine in supraventricular tachycardias, i.e. inappropriate sinus tachycardia and postural orthostatic tachycardia syndrome: HRS/ESC 2015 [13], ACC/AHA/HFSA 2016 [14], ESC 2019 [15]. ACC, American College of Cardiology; EMA, European Medicines Agency; ESC, European Society of Cardiology; FDA, US Food and Drug Administration; HRS, Heart Rhythm Society

During the study period 2007–2019, our institute received 97 requests for ivabradine, 56 (58%) were from HCP, and 41 from patients. For further details, see Fig. 1. Treatment indications for ivabradine over time in relation to formal approval status and pregnancy labelling are summarized in Fig. 2. Supraventricular tachycardia was the most common reason for ivabradine treatment in our cohort. The first ivabradine-exposed pregnancy was recorded in March 2007, 1 year after approval and marketing authorization in Germany in January 2006.

Fifty-six of the 97 requests were related to maternal exposure during pregnancy (Fig. 1). In 41 of these cases, follow-up on pregnancy outcome could be completed: 38 pregnancies were prospectively ascertained and three were retrospective reports.

### Evaluation of prospectively ascertained pregnancies

#### Maternal characteristics

The median age of pregnant women was 29.5 years and the median BMI 22.5. Maternal characteristics are presented in detail in Table 1. The mean time at first contact was gestational week (GW) 7. Only 16 of the 38 prospectively ascertained pregnancies were actually planned (42%) and only 7 women (18%) started folic acid before conception. Contraceptive methods, mostly oral contraceptives (*n* = 4), were still used by 6 women at conception.

**Table 1:** Maternal characteristics and pregnancy outcomes of ivabradine exposed pregnancies. Data are *n* (%) except for age, BMI, gestational week at birth, neonatal weight, length and head circumference, which are median and interquartile range

Maternal characteristics		*N* (%) or median (IQR)
Maternal characteristics	Age (*n=*38)	29.5 (26.5-34)
	BMI (*n=*34)	22.5 (19.4-25.6)
	Smoking (*n=*37)	
	>5 cigarettes/day	7 (19%)
	<=5 cigarettes/day	4 (11%)
	Alcohol (*n=*36)	
	<=1 drink/day	2 (6%)
	>1 drink/day	1 (3%)
	GW at first contact (*n=*38)	7 (5.6-11.1)
	Previous pregnancies (*n=*38)	
	0	17(45%)
	1	8 (21%)
	2	13 (34%)

Pregnancy outcome and neonatal characteristics		*N* (%) or median (IQR)
Pregnancy outcome (*n=*38)	Spontaneous abortion	3
	ETOP	3
	Live birth	32
Pregnancy complications (*n=*32, only live births)	Gestational diabetes	5 (16%)
	Pre-eclampsia	2 (6%)
	Cesarean section	15 (46%)
	Preterm birth (<GW 37)	3 (9%)
Neonatal characteristics (*n=*33, including one pair of twins)	GW at birth (*n=*32)	39 (37.9-39.9)
	Weight, g (*n=*32)	3300 (2935-3610)
	Length, cm (*n=*31)	50 (49-52.5)
	HC, cm (*n=*30)	35 (34-36)
	SGA (*n=*32)	2 (6%)

*BMI,* Body Mass Index; *ETOP*, elective termination of pregnancy; *GW*, gestational week; *HC*, head circumference; *IQR*, interquartile range; *SGA*, small for gestational age (<10^th^ percentile)

#### Ivabradine exposure and co-medication

The most prevalent indication for ivabradine treatment was supraventricular tachycardia (*n* = 29) followed by heart failure (*n* = 3) and coronary artery disease (*n* = 2). Other reported indications (*n* = 4) included QT prolongation and sick-sinus-syndrome, although both are regarded as contraindications for ivabradine. The median daily ivabradine dose was 7.5 mg (IQR 5–10, min–max 2.5–15, *n* = 33).

**Fig. 3:**
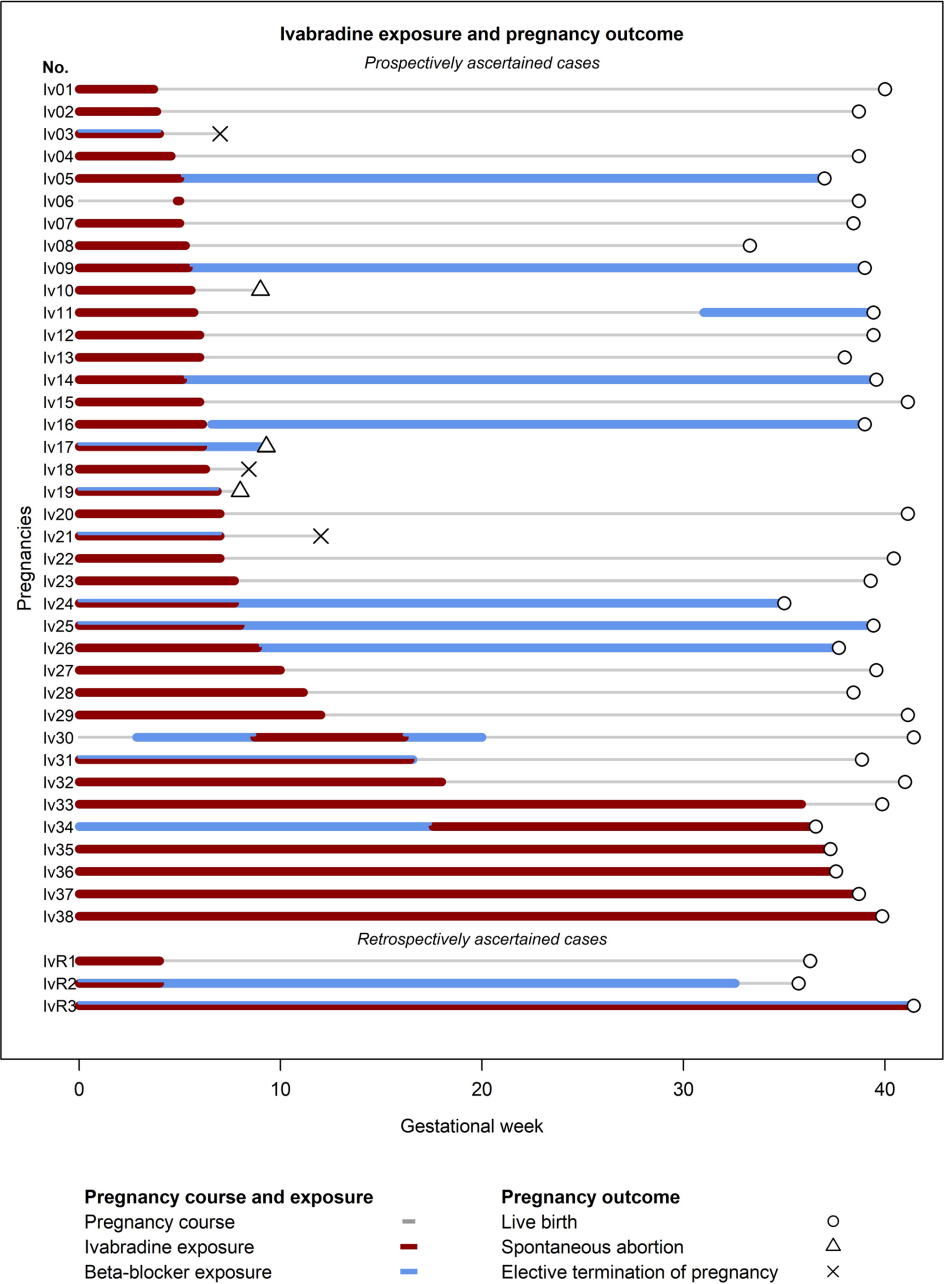
Ivabradine exposure and pregnancy outcome of prospectively (*n* = 38) and retrospectively (*n* = 3) ascertained pregnancies

Nearly all women were treated with ivabradine during the first trimester (*n* = 37) and 32 stopped treatment after recognition of pregnancy (Fig. 3), at median gestational week 6.1 (interquartile range 5.4–7.8). Cardiac decompensation or complications were not described after cessation of treatment. Only 5 women continued ivabradine throughout pregnancy or until shortly before delivery. One woman started treatment after first trimester (GW 18) of her twin pregnancy due to symptomatic tachycardia under beta-blocker therapy (Iv34 in Fig. 3).

Cardiovascular co-medication consisted of beta-blockers (mainly metoprolol) in 15 women, mostly concomitantly (*n* = 7) or in replacement of ivabradine (*n* = 6). Two women initiated ivabradine after discontinuation of beta-blockers (Iv30 and Iv34 in Fig. 3). Six women received other cardiovascular medication, three methyldopa or amlodipine for hypertension, two digitoxin or verapamil for tachyarrhythmia, and one sildenafil and eplerenone for cardiomyopathy with cardiac failure (Iv17).

Psychiatric co-medication was reported in 7/38 women (5 of these with an anxiety disorder) including the teratogenic antiepileptic topiramate (*n* = 1) and pregabalin (*n* = 3).

#### Pregnancy outcome

Of the 38 prospectively ascertained ivabradine-exposed pregnancies, 32 were live births (33 infants including one pair of twins) and three resulted in early spontaneous abortions (Iv10, Iv17, and Iv19 in Fig. 3). One of these occurred in a woman with cardiomyopathy (Iv17) and various co-medications including hydroxycarbamide for thrombocythemia until GW 7. Three pregnancies were electively terminated for social/psychological reasons. Further information on pregnancy complications and outcomes is given in Table 1 and Fig. 3.

Major birth defects were reported in one pregnancy (Iv24) with 15 mg ivabradine plus bisoprolol until GW 7 + 5, both replaced by metoprolol. Polyhydramnios and preeclampsia led to Caesarean section in GW 35 after premature rupture of membranes. The premature child was large for gestational age (> 97 percentile) and had a cleft palate, an atrial septal defect II (spontaneously closed at the age of 10 months), and a hypertrophic cardiac septum. The mother was treated for sinus tachycardia, in addition she had diabetes type I and a BMI of 30.

#### Neonatal characteristics

Characteristics of the 33 neonates are summarized in Table 1. Compared to the German perinatal survey, standardized birth weights were lower with a median SDS of − 0.36, corresponding to a reduction of birth weight of 149 g for singletons born in gestational week 40.

Two neonates were small for gestational age, one of the twins (exposure only during 2nd and 3rd trimester, Iv34) and one after maternal ivabradine exposure until GW 12 (Iv29). Two neonates were large for gestational age, both mothers were diabetic (Iv24, type I diabetes and Iv27, gestational diabetes).

### Retrospective cases

During the study period three pregnancies were retrospectively reported (further details are given in Table 2 and Fig. 3). One premature neonate (IvR1) with tracheal atresia died perinatally. The mother had been treated with ivabradine, naproxen, and metamizole during the first trimester until recognition of pregnancy (no further details available).

**Table 2:** Retrospectively reported pregnancies

Case	Maternal age/BMI	Treatment indication	Ivabradine exposure, other co-medication (GW or trimester)	Pregnancy outcome, GW, birth weight percentile, sex	Pregnancy complications, birth defects, and additional findings
IvR1	31 years/31.2	Tachycardia	Ivabradine (1st trim) Naproxen (1st trim) Metamizole (1st trim)	Live birth, 36 + 2, > 97th, f	Polyhydramnios, perinatal death, tracheal atresia, normal karyotype (46, XX)
IvR2	29 years/18.4	Tachycardia, syncopes, pacemaker	Ivabradine (1st trim) Bisoprolol (0–35 + 5) Levetiracetam (0–35 + 5, grand mal epilepsy)	Live birth, 35 + 5, < 10th, f	IUGR (since GW 30), oligohydramnios (since GW 34), Caesarean section (vaginal bleeding)
IvR3	29 years/not available	Tachycardia	Ivabradine (0–41 + 3) Bisoprolol (0–41 + 3)	Live birth, 41 + 3 > 10th, m	Uncomplicated pregnancy

*BMI*, body mass index; *f*, female; *GW*, gestational week; *IUGR*, intrauterine growth restriction; *m*, male; *trim*, trimester

The other two retrospective cases include a woman with comorbidities, intrauterine growth restriction, and premature birth (IvR2). The second was a healthy newborn (IvR3) after treatment with ivabradine throughout pregnancy. This mother reported a previous also uneventful pregnancy course under ivabradine.

## Discussion

### Pregnancy outcome

In our prospective cohort, there was only one neonate with major birth defects (cleft palate and atrial septal defect) from a diabetic mother with supraventricular tachycardia. No further major or cardiac malformations were reported among the 32 live births and 6 pregnancy losses. Cardiac defects have been observed in preclinical experimental studies. As a possible teratogenic mechanism, ivabradine-induced embryonic heart rate reduction leading to reduced cardiac output and hypoxia was discussed [16]. With a prevalence of almost 1 of 100 cardiac malformations belong to the most frequent birth defects [17, 18]. In our case, the neonate was large for gestational age and had a septum hypertrophy, supporting the assumption that maternal pre-gestational diabetes may also be causative for the observed cardiac defect and cleft palate. A causal relationship between ivabradine and cleft palate is unlikely, because ivabradine was discontinued at GW 7 + 5 before the beginning of the critical time period for the development of cleft palate at gestational week 9. The rate of major birth defects in our prospective cohort is comparable to the expected background rate of 2–3% [17, 18]. However, the limited number of pregnancies exposed during the entire period of embryogenesis does not allow excluding an increased risk, so far.

There was only one retrospectively reported case with a birth defect (malformation of the trachea) suspected as potentially ivabradine related. The concomitant exposures with naproxen and metamizole have not been evidenced as teratogens in human, whereas the high maternal BMI in the present case poses a risk for birth defects.

Taken together, we did not observe a specific pattern of congenital anomalies in our study. In addition, the presence of maternal comorbidities like diabetes and high BMI may have contributed to the observed birth defects.

The number of spontaneous abortions (3/38) was not higher than expected. The rate of preeclampsia and preterm birth in our cohort was within the expected range [19].

### Treatment indications

Although the approved treatment indications for ivabradine, chronic heart failure and chronic stable angina pectoris (see Fig. 2 for changes of licenced use during the study period), are rare in women of reproductive age, we have observed an increasing need for information for off-label use in (younger) women considering pregnancy.

In our case series, most women were treated (off-label) for supraventricular tachycardia using lower doses than recommended (5–7.5 mg twice daily). Supraventricular tachycardia is not uncommon, especially in younger women, and is expected in 0.02–0.5% of pregnancies [20]. Aggravation of symptoms during pregnancy is described in about 20–50% of women related to various mechanisms, including pregnancy-related increase in cardiac heart rate and output and hormonal changes [20].

In 2015, the HRS (Heart Rhythm Society) considered ivabradine as promising for treating patients with inappropriate sinus tachycardia (IST) [13], followed by a recommendation for ivabradine by the ACC (American College of Cardiology) in 2016 [14]—though only one small randomized controlled study has yet supported beneficial effects [21]. Although still not approved for this indication, recent ESC guidelines [15] also recommend ivabradine alone or in combination with a beta-blocker in symptomatic patients with IST, for the treatment of focal atrial tachycardia or for postural orthostatic tachycardia syndrome (POTS), a condition considered as common cause for orthostatic intolerance in women between 15 and 25 years [20, 22] (Fig. 2).

Beta-blockers were required in 18% of women in our prospective cohort (Fig. 3). Discontinuation of ivabradine or switching to beta-blockers after recognition of pregnancy was well tolerated in most of our patients. Thus, necessity of ivabradine treatment should be critically considered in women with supraventricular tachycardia planning pregnancy. Data from previous studies suggest that discontinuation of ivabradine is possible in many (non-pregnant) patients with IST without recurrence. Even beneficial long-term effects after cessation of therapy are discussed [23, 24].

A high number of women in our cohort received therapy for anxiety and/or other psychiatric conditions. This is in line with findings from other studies reporting high rates of anxiety (25%) and depression (26%) in IST patients [25]. Anxiety is considered as non-cardiac symptom of inappropriate sinus tachycardia, and vice versa, anxiety may trigger tachycardia. Especially during pregnancy, empathic care and reassurance are necessary, to prevent increased concerns about possible harm to the fetus [26].

Preconception counselling is of utmost importance in all women considering pregnancy under medication [27]. Sinus tachycardia is a condition diagnosed mainly in young females and the first diagnosis is frequently made during or after pregnancy [25]. Considering the increasing off-label use of ivabradine as well as the high rate of > 40% unplanned pregnancies [28], more exposed pregnancies can be expected in the future. Well-established treatment options such as selected beta-blockers with low reproductive risk [29] should be considered in women of childbearing age before prescription of ivabradine.

### Strength and limitations

A major strength of our study is the detailed exposure protocol and the multi-source ascertainment of pregnancy outcome via patients and their HCP followed by a case by case plausibility test.

Our data may not be representative of the German pregnant population; insofar, women with higher education are over-represented [30]. However, the regional distribution of enrolled patients is representative of the female population of childbearing age in Germany [11]. In addition, women on long-term ivabradine with severe cardiovascular conditions such as the approved treatment indications coronary artery disease and cardiac failure are under-represented in our cohort possibly favouring uneventful pregnancy outcomes. However, given the rarity of severe cardiac conditions in pregnant women, large study cohorts are not to be expected in the foreseeable future.

## Conclusions

Our findings do not indicate that ivabradine is a major teratogen when used in early pregnancy. Although being the largest study published so far on the outcome of ivabradine-exposed pregnancies, the sample is too small to rule out embryotoxic effects. Further studies are needed to confirm or refute our findings.

In women planning pregnancy, established drugs of choice with low risk for the unborn should be preferred to ivabradine. For women with significant arrhythmias and severe cardiac disease, the impact of the underlying medical condition including comorbidities such as diabetes and high BMI on pregnancy outcome has to be considered when planning pregnancy.

After inadvertent ivabradine exposure during pregnancy or lack of treatment alternatives, fetal ultrasound for structural anomalies and growth restriction is recommended. In addition, all pregnant women with complicated arrhythmias or major cardiac disease should be carefully monitored.
